# MiR-7 Regulates Pathogen-Induced Immune Response *via* PAK1 in the Sea Cucumber *Apostichopus japonicus*

**DOI:** 10.3389/fimmu.2022.927796

**Published:** 2022-07-14

**Authors:** Tanjun Zhao, Liyuan Ren, Chengda Li, Li Liu, Yang Zou, Hanyu Yan, Yaoyao Zhan, Yaqing Chang

**Affiliations:** ^1^ College of Life Science, Liaoning Normal University, Dalian, China; ^2^ Key Laboratory of Mariculture & Stock Enhancement in North China’s Sea, Ministry of Agriculture and Rural Affairs, Dalian Ocean University, Dalian, China

**Keywords:** MiR-7, PAK1, *Vibrio splendidus*, *Apostichopus japonicus*, innate immune regulation

## Abstract

MicroRNA-7 (miR-7) is a highly conserved short non-coding RNA involved in various bioprocesses *via* the regulation of multiple target genes. To enrich our knowledge of the functions of miR-7 in innate immune regulation in echinoderms, we first investigated the targeting relationship between miR-7 and *PAK1* in the sea cucumber *Apostichopus japonicus* and then explored the functions of miR-7, the *PAK1* gene, and the miR-7/*PAK1* axis in the pathogen-induced immune response of *A. japonicus*. Our results showed that miR-7 can bind to the 3ʹUTR of *PAK1* and negatively regulate the expression of *PAK1* in *A. japonicus*. Overexpression and inhibition of miR-7 and inhibition of the expression of *PAK1* can alter phagocytosis, cellular agglutination, and lysozyme contents in *A. japonicus*. Both miR-7 and the *PAK1* gene are involved in immune defense against *Vibrio splendidus* infection; the miR-7/*AjPAK1* axis showed immune regulatory function at 48 to 72 h post-infection (hpi) after *V. splendidus* infection in *A. japonicus.* In summary, the results of this study established that miR-7 regulates the pathogen-induced immune response by targeting *PAK1* in *A. japonicus*.

## Introduction

MicroRNAs (miRNAs) belong to the class of small endogenous non-coding RNAs (ncRNAs) ubiquitously expressed in metazoan organisms (1). Typically, miRNAs can directly pair with the 3’untranslated regions (3’UTR) of target genes to degrade target gene mRNA or inhibit translation of the target gene, thereby achieving posttranscriptional regulation of target gene expression (2, 3). It has been well documented that miRNAs play critical roles in many biological processes such as ontogenesis (4), sexual differentiation and maturation (5, 6), tumorigenesis (7), and immune defense (3, 8).

As an evolutionarily conserved miRNA, microRNA-7 (miR-7) is 23 nt in length, including a seed region of “-GGAAG-”. Functionally, miR-7 has been studied in both vertebrates and invertebrates. In vertebrates, miR-7 acts as an oncomir (or tumor suppressor) in several types of cancers by targeting genes associated with multiple signal pathways (9–11). In addition, miR-7 can regulate sex differentiation and maturation in pigs (12) and fish (5). In invertebrates, miR-7 has been demonstrated to play a critical role in the immune defense response against pathogen infection in crustaceans (13, 14). In recent years, increasing numbers of target genes of miR-7 have been identified; however, there are still many target genes of miR-7 that have not been identified or investigated.

The sea cucumber *Apostichopus japonicus* (Holothuroidea, Echinodermata) is not only a fishery species of economic importance in Asian countries (15) but is also an excellent model organism for inferring the evolution of innate immunity (16). The target genes of miR-7 involved in regulating the pathogen-induced immune response of *A. japonicus* are largely unknown. There is only one transcriptomic study predicting that miR-7 may regulate pathogen-induced innate immune reactions in *A. japonicus* by targeting lipopolysaccharide-induced tumor necrosis factor-alpha factor-like (17). It is worth noting that our bioinformatic prediction data showed that there may be a potential regulatory targeting relationship between miR-7 and *PAK1* (*p21-activated kinase 1*) in *A. japonicus*. Since we previously identified *PAK1* of *A. japonicus* (*AjPAK1*) and preliminarily demonstrated its immune-related function (16), we herein hypothesized that miR-7 might regulate the innate immunity of *A. japonicus via* targeting *AjPAK1*.

To clarify the function of miR-7 and the relationship between miR-7 and *AjPAK1* in regulating the pathogen-induced immune response of *A. japonicus*, in the current study we first validated the targeting relationship between miR-7 and *AjPAK1* and then investigated the expression alteration of miR-7 and AjPAK1 (at both mRNA and protein levels) after *Vibrio splendidus* (the major pathogen of skin ulceration syndrome in sea cucumbers) infection. Moreover, we investigated alterations in phagocytic capacity, cellular agglutination, and lysozyme contents in *A. japonicus* after infection with *V. splendidus*. Finally, we investigated the functional model of the miR-7/*AjPAK1* axis in regulating the pathogen-induced immune response of *A. japonicus*. The results of this study could further enrich our knowledge of the miR-7 function and its targeting networks in the innate immunity regulation of sea cucumbers.

## Materials and Methods

### Experimental Animals and Cell Culture

Healthy specimens of *A. japonicus* (average wet body weight 120 ± 13 g) were provided by the Yinhaima aquaculture (Dalian) Co., Ltd (Liaoning province, China). Prior to experimentation, all specimens were maintained in ~1000 L laboratory circulating seawater tanks without feeding for one week. During the temporary incubation, freshly filtered seawater (FSW) was maintained at 15 ± 0.5°C, with a salinity of 31 ± 0.34 and a pH of 8.04 ± 0.03.

HEK-293T cells were purchased from the China Center for Type Culture Collection (CCTCC) and maintained in DMEM (Gibco, USA) supplemented with 10% fetal bovine serum (FBS) cultured at 37°C in a 5% CO_2_ incubator.

### Sample Collection for cDNA Isolation and Expression Profiling

We selected nine healthy adult *A. japonicus* (three groups of three individuals) and starved them for 3 days. We then carefully removed samples of the following tissues: tube foot, coelomocytes, body wall, intestine, respiratory tree, and longitudinal muscle. For the collection of coelomocytes, we collected coelomic fluid and centrifuged it immediately at 3000 rpm for 15 min at 4°C. All samples were immediately frozen in liquid nitrogen and stored at −80°C.

### Total RNA Extraction and cDNA Synthesis

Total RNA was extracted from each sample using TRIzol (Ambion, USA) in accordance with the manufacturer’s instructions. Total RNA quantity and integrity were assessed using 1% agarose gel electrophoresis and an RNA Nano 6000 assay kit with an Agilent Bioanalyzer 2100 system (Agilent Technologies, CA, USA) (16). Primer Premier v5.0 (Premier Biosoft, Canada) was used to design the specific primers for *AjPAK1* (**Table 1**).

**Table 1:** The PCR primers used in this study.

Primer	Sequence (5’-3’)	Application
*AjPAK1-F*	TTGAGATGATTGAGGGGGAAC	qRT-PCR
*AjPAK1-R*	CAGAAAACTTTTGAAGACGGG	qRT-PCR
*CYTB-F*	TGAGCCGCAACAGTAATC	Reference gene
*CYTB-R*	AAGGGAAAAGGAAGTGAAAG	Reference gene
miR-7	CGCGTGGAAGACTAGTGATTTTGTTGT	qRT-PCR
RNU6B	ACGCAAATTCGTGAAGCGTT	Reference gene

### Spatial Expression Analysis by qRT-PCR and Western Blotting

We performed qRT-PCR on an Applied Biosystem 7500 Real-time System (Applied Biosystems, MA, USA) to analyze the gene expression profiles of miR-7 and *AjPAK1* (see **Table 1** for the primers used). The qRT-PCR was performed in a 20-μL reaction sample containing 2 μL of cDNA, 10 μL of 2× TB Green *Premix Ex Taq II* (Tli RNaseH Plus), 0.4 μL of ROX Reference DyeII, 6 μL of PCR-grade water, and 0.8 μL (10 mM) of each primer. The cycling program was as follows: 95°C for 600 s followed by 45 cycles of 95°C for 10 s, and 60°C for 60 s. At the end of the amplification, the presence of single-PCR product was confirmed *via* the PCR melting curve (16). The relative expression level was determined by the comparative 2^-ΔΔCt^ method (18).

The recombinant expression of AjPAK1 protein and the preparation of polyclonal antibody were completed by Dia-An Biotechnology (Wuhan, China). The experimental steps of Western blotting were as follows. The tissue samples were lysed in 300 μL RIPA lysis buffer (Biosharp, China). A BCA protein detection kit (Beyotime, Shanghai, China) was used to determine the concentration of each protein sample, and the specific detection method was carried out according to the manufacturer’s instructions. Total tissue protein was separated on 10% SDS-PAGE gels, and the proteins were electrophoretically blotted to nitrocellulose filter (NC) membranes (Biosharp, China). Then, the membranes were blocked with 5% non-fat-powered milk (Sangon Biotechnology, Shanghai, China) in TBST buffer (100 mM NaCl, 100 mM Tris-HCl, 0.05% Tween-20, pH 7.5) for 12 h. The blocked membranes were incubated in TBST buffer containing 5% non-fat-powered milk with rabbit AjPAK1 (100:1) and β-tubulin antibody (Abgent, China; 5000:1) at 15°C for 4 h. The membranes were washed in TBST buffer five times for 5 min each and then incubated with 1:5000 HRP-conjugated goat anti-rabbit IgG (Sangon Biotech, China) at 15°C for 2 h. After washing three times for 5 min each in TBST buffer, the detection was performed using Immobilon Western Chemilum HRP Substrate (Merck Millipore, Germany) with an Amershan Imager 600 (GE, USA). The grey value of each band was measured using Image-Pro Plus 6.0 (Media Cybernetics, USA). To test the specificity of the AjPAK1 antibody, the antiserum was preabsorbed with the purified recombinant AjPAK1 protein at 4°C for 16 h. As a negative control, the preimmune serum from the same rabbit was subjected to Western blot detection.

### Target miRNA Prediction and 3’-UTR Luciferase Reporter Assay

A miR-7 binding site was obtained by analyzing the AjPAK1 3’UTR sequence using RNA22 software. The 3’-UTR of wild-type (WT) *AjPAK1* mRNA containing one putative target site of miR-7 and one kind of 3’-UTR mutant-type (MUT) *AjPAK1* mRNAs (*AjPAK1* 3’-UTR MT) were synthesized by Sangon Biotechnology (Shanghai, China) and cloned into the pmirGLO luciferase plasmid (Promega) between the SacI and SalI restriction sites. These clones were further confirmed by sequencing (Sangon Biotechnology, Shanghai, China). For the transfection experiment, HEK-293T cells were seeded into a 96-well white TC plate with a total volume of 100 μL. Two solutions were prepared in each well as follows: the first solution contained 200 ng of pmirGLO luciferase plasmid or pmirGLO luciferase plasmid containing either the wild-type or mutated *AjPAK1* 3’-UTR and 0.5 μL lipofectamine 2000 (Lip 2000; Invitrogen, USA) transfection reagent. The second solution comprised 50 nM of miR-7 mimic and 0.5 μL of Lip 2000. Subsequently, 25 μL of each solution were mixed and incubated at room temperature for 20 min. The solutions were then replaced by 50 μL of a medium in each well. At 48 h post-transfection, the cells were collected for activity determination using the Dual-Luciferase Reporter Assay System (E1980, Promega). Luciferase activity was calculated based on the luciferase signal ratio by SpectraMax i3x (Molecular Devices, USA) between the Firely Luciferase and Renilla Luciferase. All of the experiments were performed in three replicates. Relative luciferase activity was normalized to Renilla Luciferase.

### Functional Analysis of miRNA *In Vivo*

The miRNA agomir (miR-7 overexpression), antagomir (miR-7 inhibition), and negative control (NC) were designed and synthesized at Gene-Pharma (Shanghai, China; **Table 2**) and were dissolved in RNase-free water to obtain a working solution of 20 μM. MiR-7 agomir (or antagomir) or NC (10 μL) were with mixed with 10 μL of Lip 2000 transfection reagent and 80 μL of PBS to serve as the transfection solution. Healthy sea cucumbers (average wet body weight 50 ± 13 g) were injected with 100 μL of transfection solution or NC solution mixture. At 24 h post-transfection, the coelomocytes from each group were collected, immediately frozen in liquid nitrogen, and stored at −80°C for further analyses by qRT-PCR and Western blotting. The assays described above were biologically repeated three times and were run in triplicate.

**Table 2:** Synthetic sequences used in this study.

Name	Sequences (5’-3’)	Application
SiPAK1	GGAGCUCAUUAUCAAUGAATT UUCAUUGAUAAUGAGCUCCTT	SiRNA
miR-7 agomir	UGGAAGACUAGUGAUUUUGUUGU AACAAAAUCACUAGUCUUCCAUU	miR-7 overexpression
miR-7 antagomir	ACAACAAAAUCACUAGUCUUCCA	miR-7 inhibition
NC	sense: UUCUCCGAACGUGUCACGUTT; antisense: CAGUACUUUUGUGUAGUACAA	negative control

NC, negative control.

### Silencing of *AjPAK1* Using siRNA

Specific small interfering RNAs (siRNAs) targeting *AjPAK1* (RNAi) and NC were designed and synthesized by Gene-Pharma (Shanghai, China; **Table 2**). For the *in vivo AjPAK1* knockdown, 10 μL of SiPAK1 (20 μM) or NC were mixed with 10 μL of Lip 2000 transfection reagent and 80 μL of PBS to serve as the transfection solution. Healthy sea cucumbers (average wet body weight 80 ± 13 g) were injected with 100 μL of SiPAK1 mixture or the NC mixture. At 24 h post-transfection, the coelomocytes from the SiPAK1 group and control group were collected, immediately frozen in liquid nitrogen, and stored at −80°C for further analyses by qRT-PCR and Western blotting. The assays described above comprised three biological repeats and were run in triplicate.

### *V. splendidus* Infection and Sample Collection

To investigate the expression of *AjPAK1* in response to bacterial infection, *V. splendidus* (strain No.: D4501), the primary pathogen associated with sea cucumber skin ulceration disease, was chosen to infect *A. japonicus* (19). Before the infection experiment, *V. splendidus* was cultured and collected according to the method of Ren et al. (16). We selected 40 healthy adult *A. japonicus*, and randomly divided them into two groups of 20 individuals each. All specimens in the challenge group were immersed in seawater containing *V. splendidus* (1 × 10^7^ CFU·mL^−1^), and the specimens of another group without any treatment served as controls. Three individuals of each group were randomly selected at 0, 4, 8, 24, 48, 72 and 96 h post-infection (hpi). The coelomocytes from each group were collected, immediately frozen in liquid nitrogen, and stored at −80°C for further analyses by qRT-PCR and Western blotting.

### *In vitro* Analysis of Phagocytosis and Cellular Agglutination of Coelomocytes After *V. splendidus* Infection

A total of 84 healthy and vigorous *A. japonicus* were randomly divided into four groups, as shown in **Table 3**. After 24 h of treatment, the sea cucumbers of each group were immersed in seawater containing *V. splendidus* (1 × 10^7^ CFU/mL). Samples were taken at 0, 4, 8, 24, 48, 72, and 96 hpi. Three individuals were randomly selected from each group, and the coelomic fluid of each individual was collected at each sampling time point.

**Table 3:** Grouping and treatment of sea cucumbers in the phagocytic capacity experiment.

Group	Number	Injection *in vivo*
SiPAK1 group	12	10 μL SiPAK1 + 10 μL Lip 2000 + 80 μL PBS
miR-7 overexpression group	12	10 μL miR-7 agomir + 10 μL Lip 2000 + 80 μL PBS
miR-7 inhibition group	12	10 μL miR-7 antagomir + 10 μL Lip 2000 + 80 μL PBS
NC group	12	10 μL NC + 10 μL Lip 2000 + 80 μL PBS

NC, negative control; Lip 2000, Lipofectamine 2000; PBS, phosphate buffer saline.

#### Phagocytic Capacity Analysis

The coelomic fluid (5 mL) was combined with the same amount of conditioned yeast suspension according to the method of Tian et al. (20) at each time point after *V. splendidus* infection, 50 μL of the mixed solution was taken to observe the phagocytosis at 60 min after the reaction based on the method of Liu et al. (19). Under an optical microscope (Leica, Germany), we recorded the number of phagocytes in different states and calculated the phagocytic capacity according to the following formula:


$$\begin{array}{l} \text{Phagocytic capacity}\left(\%\right)=(\text{total number of phagocytes extending pseudopod close to yeast}+\\ \text{total number of phagocytes phagocytizing yeast})\;/\text{ total number of phagocytes counted }\times \;100\% \end{array}$$

#### Cellular Agglutination Measurement

Coelomic fluid (2 mL) was taken in a petri dish (diameter 2 cm), and the body cavity cells were then allowed to agglutinate naturally. After 45 min, the agglutination area was observed and calculated by Image-Pro Plus 6.0 (Media Cybernetics, USA).

### Lysozyme Content Determination

Coelomic fluid (0.2 mL) was taken in a 10 mL centrifugal tube, and lysozyme (LZM) content was determined by the blank control method using a commercial kit (Jian Cheng, Nanjing, China) (**Table 4**). After the reagent was added and mixed, the sample was bathed in water at 37°C for 15 minutes, then immediately bathed in ice water for three minutes, after which the transmittance was measured at 530 nm.

**Table 4:** Operation table for determination of lysozyme content.

Reagents	Blank group	Standard group	Test group
Double distilled water (μL)	200	−	−
Standard application liquid (μL)	−	200	−
Samples (μL)	−	−	200
Standard bacteria liquid (μL)	2000	2000	2000

The LZM content per mL of the sample was calculated by the following formula:


$$\begin{array}{l} \text{LZM content}\left(\mu \text{g}/\text{mL}\right)=\text{ [}\left(\text{Test group transmittance }-\text{ Blank group transmittance}\right)\;/\;\\ \left(\text{Standard group transmittance }-\text{ Blank group transmittance}\right)\text{] }\times \;2.5\;\times \text{ Sample dilution ratio}. \end{array}$$

### Statistical Analysis

All data were expressed as mean ± standard deviation (S.D.). Data statistical significance for comparisons of miR-7 expression, AjPAK1 (mRNA and protein) expression, phagocytic capacity, cellular agglutination, and LZM content levels between two groups or among more than two groups were analyzed by independent-sample *t* tests or one-way analysis of variance (ANOVA) in SPSS v25.0 (www.spss.com), respectively. We considered *P* < 0.05 significant and *P* < 0.01 as extremely significant.

## Results

### Spatial Alterations in miR-7 and AjPAK1 (mRNA and Protein) Expression

**Figure 1:**
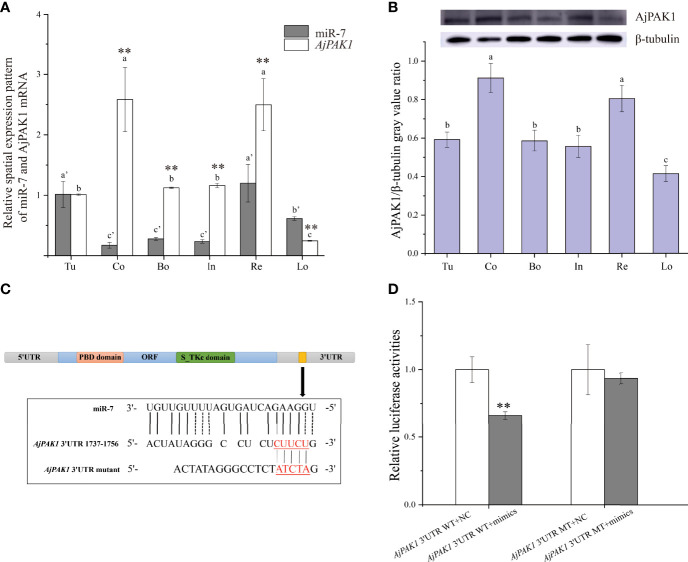
Spatial expression patterns of miR-7 and AjPAK1 (mRNA and protein) in *Apostichopus japonicus* and identification of miR-7 binding sites in the 3’UTR of *AjPAK1*. **(A)** Relative spatial expression patterns of miR-7 and *AjPAK1* mRNA. **(B)** Relative spatial expression pattern of AjPAK1 protein. **(C)** The predicted target site of miR-7 was found in the 3’UTR of *AjPAK1.* **(D)** Analysis of relative luciferase activities. u, tube foot; Co, coelomocytes; Bo, body wall; In, intestine; Re, respiratory tree; Lo, longitudinal muscle.. ** represents an extremely significant difference (*P* < 0.01) between *AjPAK1* mRNA and miR-7 in the same tissue or between *AjPAK1* 3’UTR WT + NC and *AjPAK1* 3’UTR WT + mimics. Different lower-case letters (e.g., a or a’) indicate significant differences between different tissues (*P* < 0.05).

Both miR-7 and *AjPAK1* exhibited a tissue-specific spatial expression pattern; miR-7 was highly expressed in the tube foot and respiratory tree followed by the longitudinal muscle, and the relative expression of miR-7 in the coelomocytes, body wall, and intestine was significantly lower than in other tissues (**Figure 1A**). Both AjPAK1 mRNA and protein were detected in all examined tissues, and the relative expression levels in coelomocytes and the respiratory tree were significantly higher than in the body wall, longitudinal muscle, intestine, and tube foot (**Figures 1A, B**).

### Validation of the Targeting Relationship Between miR-7 and *AjPAK1* Through Dual-Luciferase Reporter Assays

To fully validate the binding sites of miR7 at the 3’-UTR of *AjPAK1* predicted previously, luciferase reporter vectors containing WT and MT fragments of the *AjPAK1* 3’-UTR were constructed (**Figures 1C, D**). The results showed that the WT 3’-UTR + miR-7 mimics group had a remarkably lower luciferase activity than the negative control groups (**Figure 1D**). This observation indicated the existence of binding sites between miR-7 and *AjPAK1*, confirming the target relationship between miR-7 and *AjPAK1*.

### Expression of miR-7 and AjPAK1 (mRNA and Protein) After *V. splendidus* Infection

**Figure 2:**
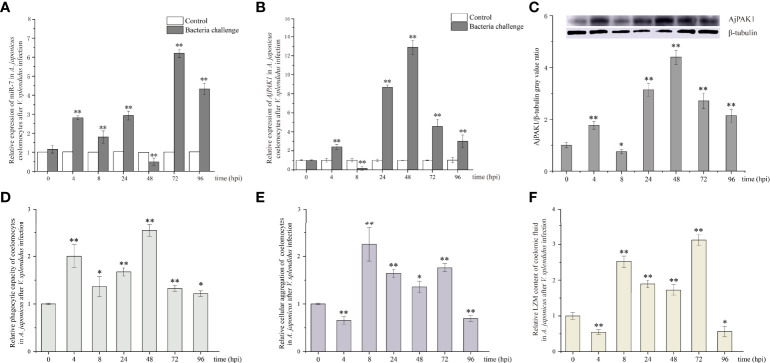
Relative expression of miR-7 and AjPAK1 (mRNA and protein) and phagocytic capacity, cellular agglutination, and lysozyme (LZM) content analyses in *A. japonicus* after *Vibrio splendidus* infection. **(A)** Relative expression of miR-7 in *A. japonicus* coelomocytes after *V. splendidus* infection. **(B)** Relative expression of *AjPAK1* in *A. japonicus* coelomocytes after *V. splendidus* infection. **(C)** Relative expression of AjPAK1 protein in *A. japonicus* coelomocytes after *V. splendidus* infection. **(D)** Relative phagocytic capacity of coelomocytes in *A. japonicus* after *V. splendidus* infection. **(E)** Relative cellular agglutination of coelomocytes in *A. japonicus* after *V. splendidus* infection. **(F)** Relative LZM content of coelomic fluid in *A. japonicus* after *V. splendidus* infection. ** represents an extremely significant difference compared with 0 hpi (*P* < 0.01); * represents a significant difference compared with 0 hpi (*P* < 0.05).

The relative expression trends of both miR-7 and AjPAK1 mRNA exhibited fluctuation in the coelomocytes of *A. japonicus* after *V. splendidus* infection. Specifically, the relative expression of miR-7 was significantly increased at 4, 8, 24, 72, and 96 hpi (*P* < 0.01) (**Figure 2A**) and significantly decreased at 48 hpi (*P* < 0.01) (**Figure 2A**) compared with that of the control at the same time points. The relative expression of AjPAK1 (mRNA and protein) was significantly increased at 4, 24, 48, 72 and 96 hpi (*P* < 0.01) (**Figures 2B, C**) and significantly decreased at 8 hpi (*P* < 0.01) (**Figures 2B, C**) compared with that of the control at the same time points. Opposite expression trends between miR-7 and AjPAK1 mRNA were observed within 8 to 72 hpi (**Figures 2A, B**).

### Changes in Phagocytic Capacity, Cellular Agglutination, and Lysozyme (LZM) Content in *A. japonicus* after *V. splendidus* Infection

The relative expression trends of phagocytic capacity, cellular agglutination, and LZM content exhibited fluctuations in the coelomic fluid of *A. japonicus* after *V. splendidus* infection. Specifically, the phagocytic capacity was significantly increased at 4, 8, 24, 48, 72 and 96 hpi (*P* < 0.05) (**Figure 2D**); both the cellular agglutination and LZM content were significantly increased at 8, 24, 48 and 72 hpi (*P* < 0.01) (**Figures 2E, F**) and significantly decreased at 4 and 96 hpi (*P* < 0.01) (**Figures 2E, F**) compared with the 0 hpi.

### Functional Analysis of miR-7 and RNA Interference Analysis of AjPAK1 *in vivo*

**Figure 3:**
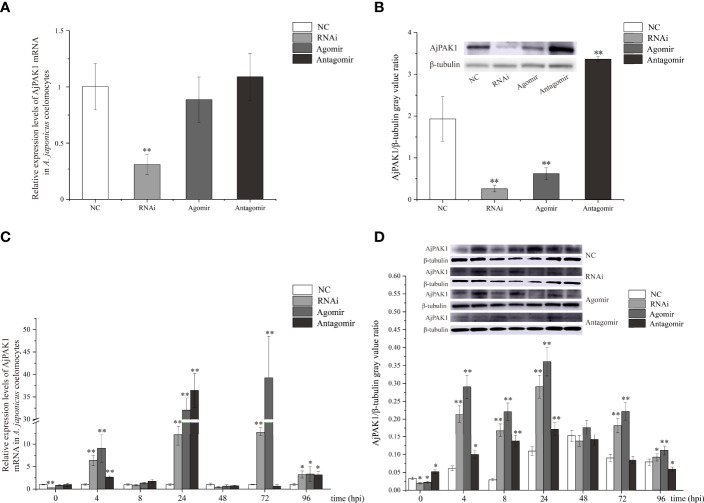
Relative expression of *AjPAK1* in coelomocytes of *Apostichopus japonicus*. **(A)** Relative expression levels of *AjPAK1* mRNA in *A. japonicus* coelomocytes after *AjPAK1* silencing, miR-7 overexpression, or miR-7 inhibition. **(B)** Expression levels of AjPAK1 protein in *A. japonicus* coelomocytes after *AjPAK1* silencing, miR-7 overexpression, or miR-7 inhibition. **(C)** Relative expression levels of AjPAK1 mRNA in *A. japonicus* coelomocytes after *AjPAK1* silencing, miR-7 overexpression, or miR-7 inhibition upon *Vibrio splendidus* infection. **(D)** Expression levels of AjPAK1 protein in *A. japonicus* coelomocytes after *AjPAK1* silencing, miR-7 overexpression, or miR-7 inhibition upon *V. splendidus* infection. NC, negative control; RNAi, *AjPAK1* silencing; Agomir, miR-7 overexpressed; Antagomir, miR-7 inhibition. ** represents an extremely significant difference compared with NC or 0 hpi (*P* < 0.01); * represents significant difference compared with NC or 0 hpi (*P* < 0.05).

After verifying that miR-7 and *AjPAK1* had binding sites, *in vivo* function verification experiments were carried out to further verify the targeted regulation relationship. Decreased relative expression of AjPAK1 mRNA was only observed in the RNA interference (RNAi) group, and no significant differences were detected in relative expression levels of AjPAK1 mRNA either in the miR-7 overexpression (agomir) group or the miR-7 inhibition (antagomir) group, whereas the Western blot results showed that altered AjPAK1 protein relative expression levels were obtained in the RNAi group, miR-7 overexpression (agomir) group, and miR-7 inhibition (antagomir) group (**Figures 3A, B**). We found that when miR-7 was overexpressed and *AjPAK1* was silenced, the relative expression level of AjPAK1 protein decreased significantly (*P* < 0.01), while when the expression of miR-7 was inhibited, the relative expression level of AjPAK1 protein increased significantly (*P* < 0.01) (**Figure 3B**). These observations indicate that miR-7 negatively regulates AjPAK1 protein expression from a posttranscriptional aspect and that siRNA of *AjPAK1* inhibits AjPAK1 protein expression at the transcriptional level.

### Functional Analysis of miR-7 and RNA Interference Analysis of AjPAK1 *In Vivo* After *V. splendidu*s Infection

After verifying that miR-7 and *AjPAK1* have a targeted regulatory relationship *in vivo*, we further investigated the relative expression of AjPAK1 (mRNA and protein) in coelomocytes under AjPAK1 silencing, miR-7 overexpression, or miR-7 inhibition after *V. splendidus* infection. The relative expression levels of AjPAK1 mRNA and protein decreased significantly (*P* < 0.01) when miR-7 was overexpressed, while the relative expression levels of AjPAK1 mRNA and protein increased significantly (*P* < 0.01) (**Figures 3C, D**) when the expression of miR-7 was inhibited or *AjPAK1* was silenced.

### Functional Analysis of miR-7 and *AjPAK1* on Phagocytic Capacity and Cellular Agglutination of Coelomocytes in *A. japonicus*

**Figure 4:**
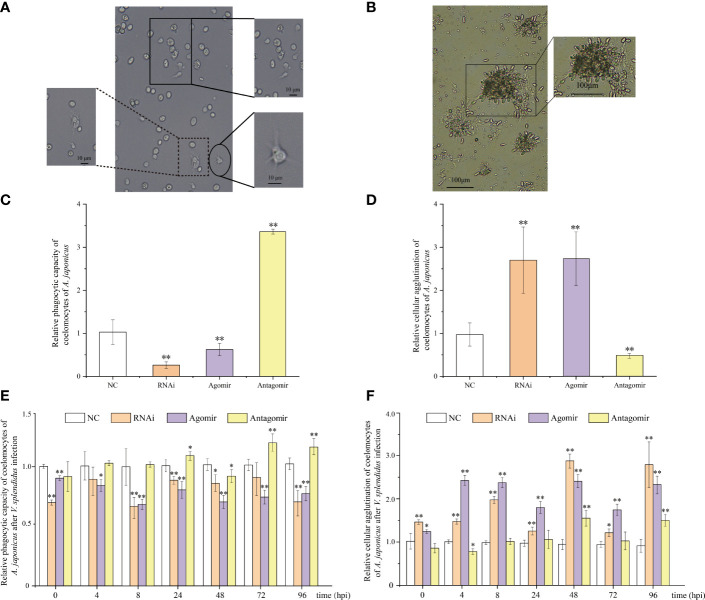
Functional analysis of miR-7 and *AjPAK1* on phagocytic capacity and cellular agglutination of coelomocytes in *Apostichopus japonicus.* **(A)** Morphology of phagocytes and the process of phagocytosis of yeast cells of *A. japonicus.* The solid box indicates that phagocytes extend pseudopods and approach yeast cells; the dotted box represents phagocytes that engulfed yeast cells; the ellipse frame indicates the normal morphology of the phagocytes. **(B)** Cellular agglutination effect of coelomocytes in *A. japonicus*. **(C)** Effects of *AjPAK1* silencing, miR-7 overexpression, and miR-7 inhibition on relative phagocytic capacity of coelomocytes of *A. japonicus*. **(D)** Effects of *AjPAK1* silencing, miR-7 overexpression, and miR-7 inhibition on relative cellular agglutination of coelomocytes of *A. japonicus*. **(E)** Effects of *AjPAK1* silencing, miR-7 overexpression, and miR-7 inhibition on relative phagocytic capacity of coelomocytes of *A. japonicus* after *Vibrio splendidus* infection. **(F)** Effects of *AjPAK1* silencing, miR-7 overexpression, and miR-7 inhibition on relative cellular agglutination of coelomocytes of *A. japonicus* after *V. splendidus* infection. NC, negative control; RNAi, *AjPAK1* silencing; Agomir, miR-7 overexpressed; Antagomir, miR-7 inhibition ** represents an extremely significant difference compared with NC (*P* < 0.01); * represents significant difference compared with NC (*P* < 0.05).

Phagocytic capacity and cellular agglutination analyses were performed to further confirm that both miR-7 and *AjPAK1* are involved in innate immune defense. The phagocytic capacity was expressed as the ratio of the sum of the number of phagocytic cells extending the pseudopod approaching yeast cells and the number of phagocytic cells phagocytosing yeast cells to the total number of phagocytic cells (**Figure 4A**). The agglutination effect was evaluated by cell agglutination area (**Figure 4B**).

Under normal conditions (without bacterial infection), when miR-7 was overexpressed or AjPAK1 was silenced, the phagocytic capacity of coelomocytes decreased significantly (*P* < 0.01), and the agglutination of coelomocytes increased significantly (*P* < 0.01), whereas when the expression of miR-7 was inhibited, the phagocytic capacity of coelomocytes increased significantly (*P* < 0.01) and the agglutination of coelomocytes decreased significantly (*P* < 0.01) (**Figures 4C, D**).

Subsequently, we investigated alterations of phagocytic capacity and agglutination in coelomocytes under *AjPAK1* silencing, miR-7 overexpression, and miR-7 inhibition after *V. splendidus* infection. In terms of *AjPAK1* silencing (RNAi) and miR-7 overexpression (agomir) groups, coelomocyte phagocytic capacity exhibited a significantly decreased trend after *V. splendidus* infection in general compared with that of the control treatment at the same time points (*P* < 0.05) (**Figure 4E**). However, significant up-regulation of coelomocyte phagocytic capacity in the miR-7 inhibition (antagomir) group was observed at 24, 72, and 96 hpi compared with the control group at the same time points (*P* < 0.05) (**Figure 4E**). For cellular agglutination, we found that increased cellular agglutination occurred across all time points examined after *V. splendidus* infection of *A. japonicus* in both *AjPAK1* silencing (RNAi) and miR-7 overexpression (agomir) groups compared with the control group (**Figure 4F**). A gradually increasing cellular agglutination trend was observed in the miR-7 inhibition (antagomir) group, with two peaks showing statistical significance at 48 and 96 hpi (**Figure 4F**).

### Effects of miR-7 and *AjPAK1* on LZM Content in the Coelomic Fluid of *A. japonicus*

**Figure 5:**
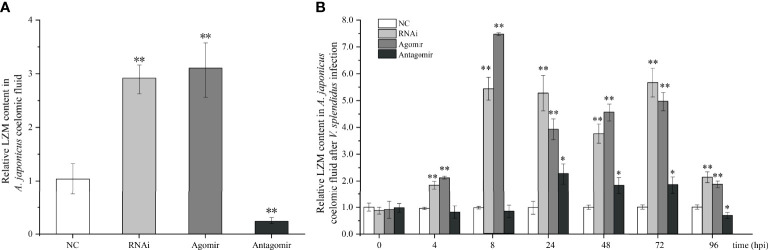
Effects of miR-7 and *AjPAK1* on relative lysozyme (LZM) content in coelomic fluid of *Apostichopus japonicus.* **(A)** Relative LZM content in *A. japonicus* coelomic fluid after *AjPAK1* silencing, miR-7 overexpression, or miR-7 inhibition. **(B)** Relative LZM content in *A. japonicus* coelomic fluid after *AjPAK1* silencing, miR-7 overexpression, or miR-7 inhibition upon *Vibrio splendidus* infection. NC, negative control; Agomir, miR-7 overexpressed; Antagomir, miR-7 inhibition. ** represents an extremely significant difference compared with NC (*P* < 0.01); * represents significant difference compared with NC (*P* < 0.05).

Under normal conditions (without bacterial infection), we found that when miR-7 was overexpressed or *AjPAK1* was silenced, the content of LZM in coelomic fluid increased significantly (*P* < 0.01), while when the expression of miR-7 was inhibited, the content of LZM in coelomic fluid decreased significantly (*P* < 0.01) (**Figure 5A**).

After *V. splendidus* infection, an increased content of LZM in coelomic fluid was observed in both the *AjPAK1* silencing (RNAi) group and the miR-7 overexpression (agomir) group compared with the control group within 4 to 96 hpi (*P* < 0.01) (**Figure 5B**). In the miR-7 inhibition (antagomir) group, a significantly increased content of LZM in coelomic fluid was observed within 24 to 72 hpi (*P* < 0.05) (**Figure 5B**), and a decreased content of LZM in coelomic fluid was observed at 96 hpi (*P* < 0.05) (**Figure 5B**).

## Discussion

In this study, the targeting relationship between miR-7 and *AjPAK1* and their functions in innate immunity in sea cucumbers were systematically clarified for the first time. Moreover, we also focused on the mechanisms by which miR-7 and *AjPAK1* regulate the immune response against pathogen infection in sea cucumbers.

The transcriptional attenuation of target genes is a conserved mechanism of miRNA regulation (21). In this study, opposite relative expression trends were observed between miR-7 and AjPAK1 (mRNA and protein) in all examined tissues, and the binding site between miR-7 and *AjPAK1* was validated, confirming the targeting relationship between miR-7 and *AjPAK1*. Moreover, we confirmed that miR-7 negatively regulates *AjPAK1* gene expression through transcriptional attenuation. This observation was not only consistent with the conserved mechanism of miRNA regulation but was also similar to the results of current gene regulation studies in sea cucumbers, for example, miR-133 and *IRAK-1* (*interleukin-1 receptor-associated kinase-1*), miR-2008 and *BHMT* (*Betaine homocysteine S-methyltransferase*), miR-137 and *BHMT*, miR-137 and *14-3-3ζ* (*14-3-3 intracellular phosphoserine/threonine-binding proteins*), miR-92a and *14-3-3ζ*, miR-2012-5p and *RhoA* (*Ras homolog A*), miR-10a-5p and *ACADL* (*Long-chain specific acyl-CoA dehydrogenase*) (3, 16, 19, 22).

The relationships of both miR-7 and *AjPAK1* with innate immunity in *A. japonicus* were the major concerns in this study. It has been well documented that immune-related genes exhibit higher relative expression trends in immune-related tissues in invertebrates (19). Consistent with our previous study (16), the spatial expression results in this study showed that the highest relative expression levels of AjPAK1 transcripts and protein were obtained in coelomocytes (the major immune-related organ of sea cucumbers) of *A. japonicus*, suggesting a possible immune-related function of AjPAK1. This observation was similar to those obtained in studies of *AjC3* (23), *MyD88*, *TRAF6* (24), *IRAK-1* (25), *p105* (25, 26), *Tollip* (27, 28), *Toll* (29), NF-κB/Rel (30), *TLR3* (17, 29), *IRAK-1* (25), *Toll* (31), *AjBHMT* (32) and *AjRhoA* (19) in *A. japonicus*. In addition, relative expression alterations were observed both in miR-7 and AjPAK1 (mRNA and protein) after *V. splendidus* infection in *A. japonicus*, indicating an involvement of both miR-7 and *AjPAK1* in response to *V. splendidus* infection. Further experimental results showed that either the knockdown of AjPAK1 expression or overexpressed miR-7 can inhibit the phagocytic capacity and enhance both cellular agglutination and LZM content. This observation indicates that the expression of miR-7 has a negative relationship with phagocytic capacity and a positive relationship with cellular agglutination and LZM content in *A. japonicus*, while the expression of AjPAK1 exhibited the opposite trend. Phagocytosis, cellular agglutination, and LZM content are considered the most basic components of the innate immune system (19, 33, 34). Activation of phagocytosis is the primary way by which the innate immune system achieves rapid elimination of exogenous pathogens (35). In phagocytosis, cells internalize particulate matter such as microorganisms, and this process is important for immune responses and during the clearance of apoptotic cells (36). Cellular agglutination is a common phenomenon that can be observed in almost all echinoderms. It was reported that the occurrence of cellular agglutination in the coelomic fluid is one of the strategies employed in response to tissue injury and pathogen infection in echinoderms (37–39). LZM is widely distributed among eukaryotes and prokaryotes. The enzyme catalyzes the hydrolysis of bacterial cell walls and acts as a nonspecific innate immunity molecule against the invasion of bacterial pathogens (33). Several studies have also reported that *RhoA* and *Rac1* can regulate phagocytosis in *A. japonicus* (19), *Marsupenaeus japonicus* (40), and *Ctenopharyngodon idella* (41), similar to the phagocytosis results for *AjPAK1* in this study. Moreover, the observation that alteration of the expression of either miR-7 or *AjPAK1* can induce changes in phagocytic capacity, cellular agglutination, and LZM content in the current study suggests that both miR-7 and *AjPAK1* may regulate innate the immune reaction from multiple perspectives in *A. japonicus*.

The core concern of this study is the mechanisms by which miR-7 and *AjPAK1* regulate the innate immune response against pathogen infection in *A. japonicus*. As we confirmed above, there is a direct negative regulation relationship between miR-7 and *AjPAK1*, and both miR-7 and *AjPAK1* were involved in the innate immune response against *V. splendidus* infection by altering phagocytic capacity, cell agglutination, and LZM content in *A. japonicus.* However, whether miR-7 and *AjPAK1* regulate the innate immune defense of *A. japonicus* alone or together is unknown. We therefore observed the dynamic progress of the *V. splendidus* infection in *A. japonicus*. As shown in the results, miR-7 and *AjPAK1* exhibited generally opposite expression trends within 8 to 72 hpi.

**Figure 6:**
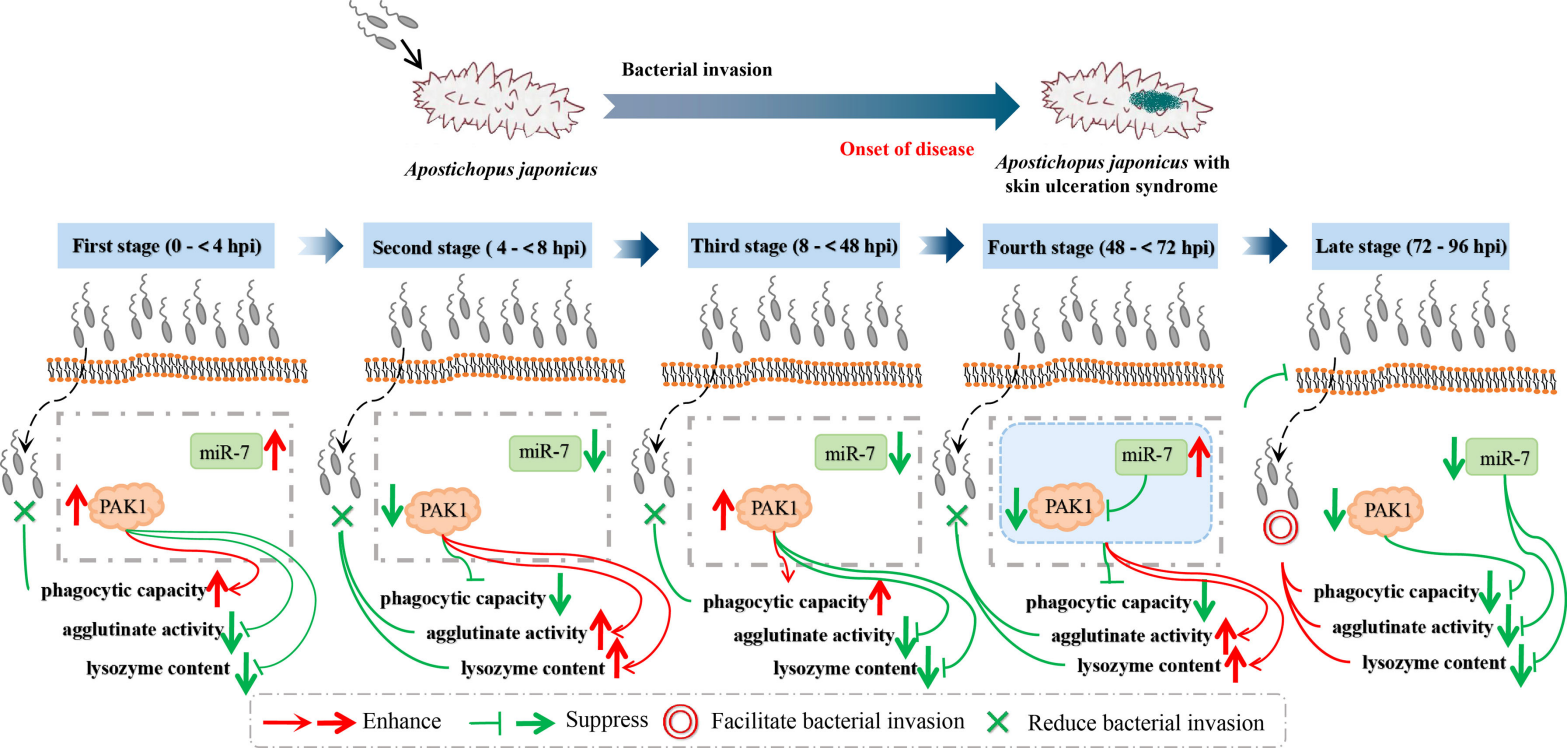
Schematic diagrams of *AjPAK1* and miR-7 involved in host-pathogen interaction of *Apostichopus japonicus*.

Specifically, at the first stages of infection (0 to < 4 hpi) and the second stage of infection (4 to < 8 hpi), the relative expression levels of miR-7 and AjPAK1 (mRNA and protein) were up-regulated first and then gradually decreased. Expression peaks and minima were observed at 4 hpi and 8 hpi, respectively. At the first stages of infection (0 to < 4 hpi), enhanced phagocytic capacity and decreased cellular agglutination and LZM content were observed, indicating that phagocytic capacity alteration is the initial strategy adopted by *A. japonicus* against pathogen infection. At the second stage of infection (4 to < 8 hpi), decreased phagocytic capacity and enhanced cellular agglutination and LZM content were observed, indicating that cellular agglutination and LZM gradually began to exert immune regulatory effects with the attenuation of phagocytic capacity in *A. japonicus* in response to pathogen infection. This observation was consistent with the AjPAK1 functions in phagocytic capacity, cellular agglutination, and LZM content (**Figures 2D–F**, **4**, and **5**) suggesting that the *AjPAK1* gene may play a major role in immune regulation in *A. japonicus* at the early stages of pathogen infection (0 to < 8 hpi) (**Figure 6**).

At the third stage of infection (8 to < 48 hpi), the relative expression of miR-7 was gradually down-regulated, reaching a minimum at 48 hpi, while the relative expression levels of AjPAK1 (mRNA and protein) were up-regulated, reaching a peak at 48 hpi. During this period, the phagocytic capacity of *A. japonicus* coelomocytes increased again, indicating enhanced bacterial elimination activity induced by up-regulation of the *AjPAK1* gene (**Figure 6**). Meanwhile, cellular agglutination and LZM content were again decreased, consistent with the AjPAK1 functions in cellular agglutination and LZM content obtained above (**Figures 2E, F**, **4D, E**, and **5**). This observation indicates that post-transcriptional attenuation did not occur in *A. japonicus* coelomocytes, although relative expression patterns of miR-7 and *AjPAK1* gene were opposite within the 24 hpi to 48 hpi period, and the *AjPAK1* gene occupied a dominant position in innate immune defense in *A. japonicus* in this period (**Figure 6**). Liu et al. reported that the miR-2012-5p/*AjRhoA* module can regulate the *V. splendidus*-induced immune response of *A. japonicus* during early infection (4 to 48 hpi) (16), combined with previously published observations that the *AjPAK1* gene alone may play a major role in immune regulation in *A. japonicus* at the early stages of pathogen infection (0 to < 48 hpi), we speculate that the genetic regulation of the innate immunity in *A. japnoicus* may be heavily time-sensitive. Further investigation is necessary.

At the fourth stage of infection (48 to < 72 hpi), the relative expression of miR-7 was gradually up-regulated, reaching a peak at 72 hpi, while the relative expression levels of AjPAK1 (mRNA and protein) were down-regulated, reaching a minimum at 48 hpi (**Figure 6**). In this period, decreased phagocytic capacity and increased cellular agglutination and LZM content were observed (**Figures 2D–F** and **6**). Alterations of phagocytic capacity and increased cellular agglutination and LZM content were consistent with the functions of miR-7 and AjPAK1 in phagocytic capacity, agglutination, and LZM content obtained above (**Figures 2D–F**, **4**, and **5**). Notably, opposite relative expression trends of miR-7 (up-regulation) and AjPAK1 (mRNA and protein; down-regulation) were observed in this period, indicating the occurrence of post-transcriptional attenuation between miR-7 and the *AjPAK1* gene; in other words, immune response alterations of *A. japonicus* coelomocytes in this period were probably due to miR-7 reducing the stability of AjPAK1 mRNA by binding to the 3’-UTR of the *AjPAK1* gene. According to this scenario, we speculated that *V. splendidus* may achieve infection *via* utilizing the miR-7/*AjPAK1* axis of the host (miR-7 inhibited the expression of *AjPAK1* in this study) directly or indirectly. The following questions were raised: what is the target of *V. splendidus*, miR-7, *AjPAK1*, or the miR-7/*AjPAK1* axis? Which signal pathway regulated by the miR-7/*AjPAK1* axis is involved in altering phagocytic capacity, cellular agglutination, and LZM content? It is clear that more detailed research work is needed in the future.

In later stage of infection (72 to 96 hpi), the relative expression levels of miR-7 and AjPAK1 (mRNA and protein) were gradually down-regulated, suggesting the completion of the miR-7/*AjPAK1* interaction. Additionally, the phagocytic capacity, cellular agglutination, and LZM content all decreased in this period, suggesting a gradually weakening bacterial elimination activity (**Figures 2D–F** and **6**). This observation also suggests a successful achievement of *V. splendidus* infection to some extent (**Figure 6**).

Combining the above observations, first, we assumed that the fluctuation of the relative expression of AjPAK1 may be partly caused by the regulation of miR-7 in the process of pathogen infection. Second, our observations support the hypothesis that the innate immune regulation network in *A. japonicus* is much more complex, and the miR-7/*AjPAK1* axis could be one of the key nodes in the innate defense network of *A. japonicus.* Third, our data partially suggest that the “mRNA-miRNA” interaction could be one of the molecular strategies in pathogen (*V. splendidus*)–host (*A. japonicus*) interactions. Furthermore, our observations provide several clues for developing disease control in sea cucumber aquaculture from the aspect of molecular immunity; by contrast, the results also suggest that more attention should be paid to developing molecular control strategies for sea cucumber disease based on the immune-related miRNA/mRNA axis.

## Conclusion

The present study validated the negative regulatory targeting relationship between miR-7 and *AjPAK1*. Both miR-7 and *AjPAK1* were involved in innate immune defense against *V. splendidus* infection by regulating phagocytic capacity, cellular agglutination, and LZM content. Specifically, miR-7 and *AjPAK1* functioned separately in innate immune defense within 0 to 48 hpi during pathogen infection, while the miR-7/*AjPAK1* axis had a regulatory role in the pathogen-induced immune response of *A. japonicus* by altering phagocytic capacity, cellular agglutination, and LZM content within 48 to 72 hpi during the infection. These findings provided references for systematically clarifying the molecular regulation mechanism of innate immune responses of invertebrates. In addition, the availability of new clues allows us to take advantage of the immune regulatory functions of specific genes and miRNA/mRNA modules to develop novel disease control strategies for economically important echinoderm aquaculture (sea cucumbers in this study) from the perspective of molecular immunity.

## Data Availability Statement

The original contributions presented in the study are included in the article/supplementary material. Further inquiries can be directed to the corresponding authors.

## Author Contributions

YYZ and YC conceived and designed the experiments. TZ, LR, LL, YZ, HY and CL performed the experiments. YYZ, TZ and YC analyzed the data. YYZ and TZ wrote the paper. All authors read and approved the manuscript.

## Funding

This study was financially supported by the National Natural Science Foundation of China (No. 32072977) and Liaoning Revitalization Talents Program (No. XLYC2002107).

## Conflict of Interest

The authors declare that the research was conducted in the absence of any commercial or financial relationships that could be construed as a potential conflict of interest.

## Publisher’s Note

All claims expressed in this article are solely those of the authors and do not necessarily represent those of their affiliated organizations, or those of the publisher, the editors and the reviewers. Any product that may be evaluated in this article, or claim that may be made by its manufacturer, is not guaranteed or endorsed by the publisher.
